# An Interferometric Synthetic Aperture Radar Tropospheric Delay Correction Method Based on a Global Navigation Satellite System and a Backpropagation Neural Network: More Suitable for Areas with Obvious Terrain Changes

**DOI:** 10.3390/s23249760

**Published:** 2023-12-11

**Authors:** Liangcai Qiu, Peng Chen, Yibin Yao, Hao Chen, Fucai Tang, Mingzhu Xiong

**Affiliations:** 1 College of Geomatics, Xi’an University of Science and Technology, Xi’an 710054, China; 21210061025@stu.xust.edu.cn (L.Q.); 21210061019@stu.xust.edu.cn (H.C.); 21210226068@stu.xust.edu.cn (F.T.); 21210061029@stu.xust.edu.cn (M.X.); 2 State Key Laboratory of Geodesy and Earth’s Dynamics, Innovation Academy for Precision Measurement Science and Technology, Chinese Academy of Sciences, Wuhan 430077, China; 3 School of Geodesy and Geomatics, Wuhan University, Wuhan 430079, China; ybyao@sgg.whu.edu.cn; 4 Key Laboratory of Geospace Environment and Geodesy, Ministry of Education, Wuhan University, Wuhan 430079, China

**Keywords:** InSAR, atmospheric correction, troposphere delay, GNSS, BP neural network

## Abstract

Atmospheric delay correction remains a major challenge for interferometric synthetic aperture radar (InSAR) technology. In this paper, we first reviewed several commonly used methods for tropospheric delay correction in InSAR. Subsequently, considering the large volume and high temporal resolution of global navigation satellite system (GNSS) station measurement data, we proposed a method for spatial prediction of the InSAR tropospheric delay phase based on the backpropagation (BP) neural network and GNSS zenith total delay (ZTD). Using 42 Sentinel-1 interferograms over the Los Angeles area in 2021 as an example, we validated the accuracy of the BP + GNSS method in spatially predicting ZTD and compared the correction effects of BP + GNSS and five other methods on interferograms using the standard deviation (StaD) and structural similarity (SSIM). The results demonstrated that the BP + GNSS method reduced the root-mean-square error (RMSE) in spatial prediction by approximately 95.50% compared to the conventional interpolation method. After correction using the BP + GNSS method, StaD decreased in 92.86% of interferograms, with an average decrease of 52.03%, indicating significantly better correction effects than other methods. The SSIM of the BP + GNSS method was lower in mountainous and high-altitude areas with obvious terrain changes in the east and north, exhibiting excellent and stable correction performance in different seasons, particularly outperforming the GACOS method in autumn and winter. The BP + GNSS method can be employed to generate InSAR tropospheric delay maps with high temporal and spatial resolution, effectively addressing the challenge of removing InSAR tropospheric delay signals in areas with significant terrain variations.

## 1. Introduction

Interferometric synthetic aperture radar (InSAR) technology is characterized by its all-weather, all-time, high-resolution, high-precision, and wide-ranging capabilities. It has been proven to be a powerful modern geodetic measurement technique in recent years [1], enabling millimeter-level observations over extensive geographical areas [2,3]. Early studies utilizing InSAR for geodetic observations achieved certain advancements [4,5,6,7]. However, more recent findings have unveiled interference patterns unrelated to surface deformation and interpreted as atmospheric delay [8,9]. Temporal and spatial variations in tropospheric temperature, pressure, and humidity result in varying degrees of delay in radar signals during repeated passes, concealing genuine surface displacements within observation outcomes [10,11]. For instance, a 20% change in relative humidity could potentially lead to a 10 cm error in deformation measurement outcomes [12]. severely restricting InSAR’s capacity to conduct high-precision observations of the Earth’s surface [13,14,15].

Over the past two decades, scholars have made numerous attempts to explore the spatial and temporal characteristics of tropospheric delay in InSAR and have conducted associated error analyses. They have sought effective means to remove tropospheric delay signals from InSAR observational outcomes [16,17]. Regarding the spatial distribution characteristics of tropospheric delay, they can be divided into vertically stratified delays correlated with terrain heights and turbulent mixed delays due to atmospheric turbulent motion [18,19]. Vertically stratified delays can be modeled as linearly correlated or as a power–law model [20,21,22,23], primarily relying on the digital elevation model (DEM) and interferometric phases for calculation [24,25]. As for the turbulent mixed delay component, often considered as spatiotemporal random noise, it can be suppressed through spatiotemporal filtering methods [26,27,28]. Since these methods typically include empirical parameters, they are collectively termed empirical approaches. Their primary advantages lie in their simplicity and speed in modeling, but they overlook the actual spatiotemporal variations in atmospheric characteristics, potentially leading to incorrect correction outcomes.

Therefore, researchers have attempted to correct the tropospheric delay in InSAR using external auxiliary data, which estimated the zenith total delay (ZTD) in the line-of-sight (LOS) direction for each SAR data acquisition moment using external auxiliary data. The commonly used external data sources fall into several categories: meteorological reanalysis data represented by ERA5 (European Center for Medium-Range Weather Forecasts Reanalysis v5) [29,30,31,32,33,34,35,36]; numerical weather forecast models represented by WRF (weather research and forecasting model) [37,38,39,40]; precipitable water vapor (PWV) products represented using MERIS (medium-resolution imaging spectrometer), Sentinel-3 OLCI (ocean and land color instrument); and MODIS (moderate-resolution imaging spectroradiometer) [41,42,43,44]. These data types originate from different sensors, and exhibit spatial continuity, clearly representing the lateral heterogeneity of tropospheric water vapor content. However, they often show varying degrees of differences in temporal acquisition and spatial resolution compared to SAR data [45]. Consequently, such methods may not be suitable for correcting atmospheric delays in areas with significant terrain changes and coastal areas.

In order to address the temporal discrepancies between external data and SAR data, scholars have proposed using GNSS station data to estimate InSAR tropospheric delay [10,46,47,48,49,50,51,52]. The limitation of this method is that the directly estimated high-time resolution ZTD only applies to the corresponding station location. Often, what we require is regional outcomes that match the interferogram. Hence, accurately interpolating the station’s ZTD to the positions of interferometric pixels becomes an inevitable challenge, particularly in areas with prominent terrain changes and higher altitudes [50]. Conventional spatial interpolation methods include global polynomial interpolation (GPI), inverse distance weighted (IDW), Kriging interpolation (KRI), and radial basis function (RBF). However, these interpolation methods do not consider the strong correlation between tropospheric delay and terrain changes. Moreover, this relationship often presents nonlinear changes with elevation within a region. Therefore, it is necessary to propose a tropospheric delay spatial prediction method that considers terrain changes and can effectively capture this nonlinearity to enhance the spatial interpolation accuracy of GNSS ZTD, ultimately addressing the challenge of removing InSAR tropospheric delay signals in regions with significant terrain changes.

In this study, we first reviewed several commonly used methods for InSAR tropospheric delay correction and briefly analyzed the advantages and disadvantages of each method. Subsequently, focusing on GNSS ZTD, we proposed a spatial prediction method for tropospheric delay based on BP + GNSS. We compared this with conventional interpolation methods to verify the accuracy of ZTD spatial prediction using the BP + GNSS method. Then, by comparing the correction effects in the Los Angeles area with other commonly used InSAR tropospheric delay correction methods, we demonstrated the outstanding and stable correction performance of the BP + GNSS method. The main advantages of this method are: (i) It can compensate for the uneven spatial distribution of GNSS ZTD, addressing the drawback of large interpolation errors in conventional methods due to non-uniform distribution. (ii) It is not affected by the low spatiotemporal resolution of other observational data. (iii) The introduction of BP neural networks can better capture the impact of tropospheric delay caused by terrain changes, further enhancing the spatiotemporal interpolation accuracy of the data. This improvement leads to better InSAR tropospheric delay correction results, particularly in areas with significant terrain changes and higher altitudes.

## 2. InSAR Tropospheric Delays

### 2.1. Components of Tropospheric SAR Delays

Tropospheric SAR delays include zenith hydrostatic delay (ZHD) and zenith wet delay (ZWD) [53]. For InSAR with repeated orbit differential interferometry, the SAR radar signals will be refracted due to the change in atmospheric refractive index during the two data acquisitions, resulting in residual atmospheric phase delays in the differential interferograms, causing confusion in the geophysical interpretation of the images. The tropospheric phase delays in single SAR data can be expressed as follows:

(1) $$\phi_{tropo\;}=-\frac{4\pi }{\lambda }\frac{10^{-6}}{cos(\theta )}\int_{h_{0}}^{h_{top\;}}\left(N_{hydr}{+N}_{wet}\right)dh$$

where λ is the radar wavelength; θ is the radar incidence angle; $h_{0}$ and $h_{top}$ denote the bottom and top layers of the troposphere, respectively; $N_{hydr}$ and $N_{wet}$ denote hydrostatic and wet delay’s atmospheric refractive index, respectively [18].

**Figure 1:**
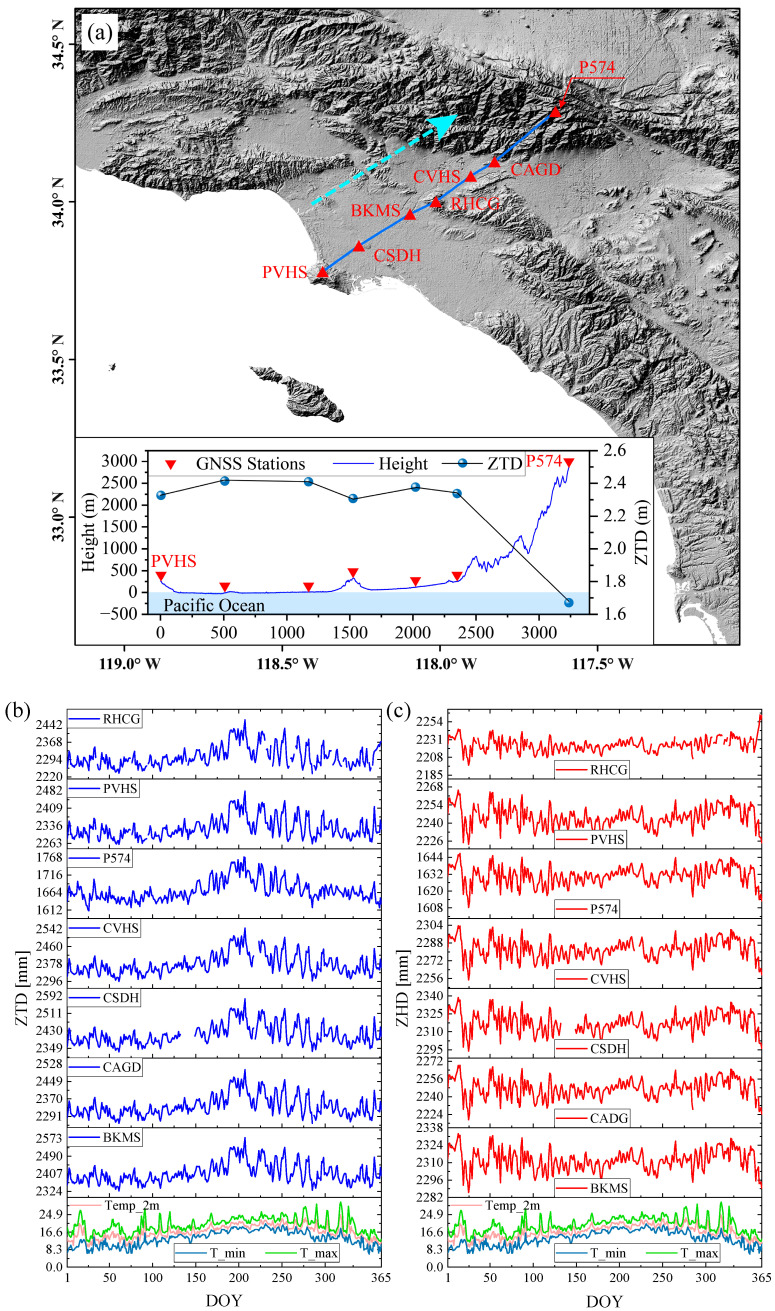
The locations of seven GNSS stations in the Los Angeles area and the ZTD and ZHD changes in the stations in 2021. The red triangles in (**a**) are selected GNSS stations, and the blue connecting lines between the stations correspond to the blue profile lines in the lower left subplot, which are oriented in the direction indicated by the dashed arrows in (**a**). (**b**,**c**) show the temperature change in Los Angeles in 2021 and the ZTD and ZHD fluctuations measured by the seven selected GNSS stations, respectively. The blue-filled background area in (**b**) is the period of significant ZTD fluctuations, and the green-filled background area in (**c**) is the period of significant ZHD fluctuations.

Figure 1 shows the annual trends of temperature, ZTD, and ZHD for seven GNSS stations in the Los Angeles region in 2021. Figure 1a shows the selected GNSS stations. The distribution of the selected stations approximates a straight line, to reflect the relationship between the ZTD value of the stations and the topography. The bottom left dashboard illustrates the negative correlation between ZTD and surface elevation. Figure 1b shows the annual fluctuation trend of ZTD, and the change in summer is greater than that in spring and winter. Figure 1c illustrates that ZHD is consistent with temperature fluctuations, with larger fluctuations in spring and winter and smaller fluctuations in summer. It indicates that the instability of ZTD in summer is caused by the significant change in ZWD, consistent with the larger tropospheric water vapor content in summer. Therefore, the InSAR tropospheric phase delays vary significantly due to the differences in the study area and time baseline. This makes it difficult to quantify the InSAR tropospheric phase delays over a long period and a large area and challenging to automate and routinely correct the interferogram.

The unwrapped phases of interferograms were obtained by differencing the SAR data obtained at two moments $t_{1}$ and $t_{2}$, which can be expressed as follows:

(2) $$\phi_{int\;}=\left(\phi_{tropo\;}^{t_{2}}-\phi_{tropo\;}^{t_{1}}\right)+\phi_{def\;}^{t_{12}}+\phi_{other\;}$$

where $\phi_{tropo}^{t_{1}}$, $\phi_{tropo}^{t_{2}}$ represent the influence of tropospheric delays at moments $t_{1}$ and $t_{2}$, respectively, $\phi_{def}^{t_{12}}$ represents the displacement of the surface in that time baseline, and $\phi_{other}$ represents other influences, such as ionospheric delays, unwrapping errors, and soil moisture.

**Figure 2:**
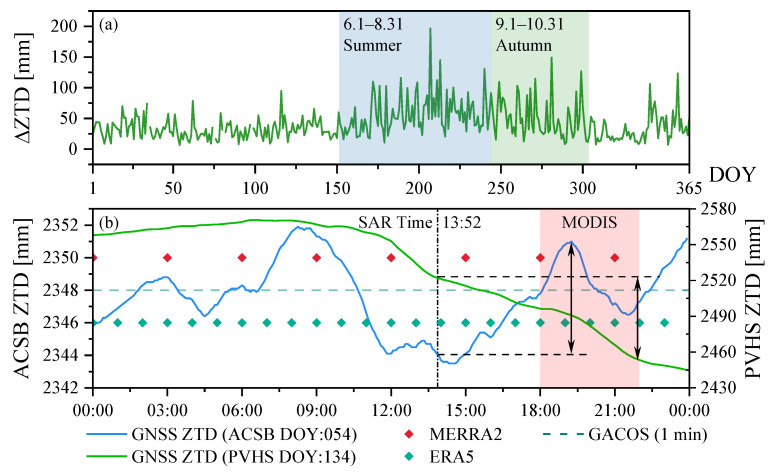
GNSS ZTD time series and time of independent data acquisitions in Los Angeles. (**a**) is the daily ZTD difference ($ZtD_{max}-ZtD_{min}$) for the GNSS station (ACSB) in 2021. (**b**) is the 24 h time series of each data in the Los Angeles region of the US. The red diamond represents the MERRA2 data with a time resolution of 3 h. The green diamond shows the ERA5 data with a time resolution of 1 h. The red background is filled with time intervals for MODIS data.

Here, we summarized the various data that can be used for InSAR tropospheric phase delay correction (Table A1). Figure 2a shows that ZTD changed significantly within 24 h of every day in 2021. The variation was especially significant in summer and September and October in autumn, with the maximum single-day difference reaching ~195 mm, and most of the variation lies within 20~100 mm. Among them, MODIS and OLCI have a significant time difference from SAR data, so the delays obtained differ from those received by SAR. There may be cases of over or insufficient correction (Figure 2b).

### 2.2. Commonly Used InSAR Tropospheric Delay Correction Methods

#### 2.2.1. Methodology Based on ZTD Product Dataset

For ZTD products given directly by the dataset, it is sufficient to project the total delay in the zenith direction of the two SAR data corresponding to each interferogram to the radar LOS direction separately, e.g., the GACOS (Generic Atmospheric Correction Online Service for InSAR) dataset [54]. The *ZTD* is converted to slant total delay (*STD*) in the radar LOS direction using Equation (3):

(3) $${STD}^{t_{i}}=\frac{{ZTD}^{t_{i}}}{cos(\theta )}\;(i=1,2)$$

where ${ZTD}^{t_{i}}$, ${STD}^{t_{i}}(i=1,2)$ are the zenith total delay and LOS direction total delay path of the two SAR data corresponding to a single interferogram, respectively.

The delayed paths are then converted into delayed phases using Equation (4):

(4) $$\phi_{tropo\;}^{t_{i}}=-\frac{4\pi }{\lambda }\times {\;STD}^{t_{i}}(i=1,2)$$

where $\phi_{tropo}^{t_{i}}\left(i\;=\;1,2\right)$ is the tropospheric phase delays of the LOS direction of the two SAR data corresponding to a single interferogram.

The obtained $\Delta \phi_{tropo}=\phi_{tropo}^{t_{2}}-\phi_{tropo}^{t_{2}}$ is then used to perform a pixel-by-pixel correction of the unwrapped phases:

(5) $$\phi_{{corr}_{int\;}}=\phi_{int}-\Delta \phi_{tropo\;}$$

The advantage of such methods is that they are fast and straightforward. The dataset can provide tropospheric delays synchronized with SAR data, avoiding the complicated steps of calculating ZTD data using other products, significantly reducing the calculation workload of the phase delays, and reducing the difficulty of InSAR tropospheric delay correction work. Its limitation is that the correction effect is not obvious in regions with large spatial spans, complex terrain changes, and sparse GNSS stations.

#### 2.2.2. Methodology Based on GNSS ZTD

ZTD is a byproduct of the GNSS solution. It can be used for at-site verification of ZTD calculated by other methods and point-by-point correction of InSAR tropospheric phase delays by interpolating its discrete station data to interferometric pixel positions. Due to the high temporal resolution and persistence of GNSS data, it is largely unaffected by the temporal fixation of SAR data. The spatial interpolation of the GNSS ZTD is performed, and the unwrapped phases are corrected according to Equations (3)–(5).

The advantage of this method is that the temporal resolution is high and continuous, while the accuracy of its ZTD data is guaranteed, and the data are acquired independently. The limitation is that the acquired ZTD data are spatially discrete. Due to global GNSS station deployment, the sparse distribution of GNSS stations in individual areas may lead to obvious errors in the interpolated data. Therefore, a GNSS ZTD spatial prediction method that can adapt to complex terrain changes is necessary.

#### 2.2.3. Methodology Based on Meteorological Reanalysis Data

For meteorological reanalysis data (such as ERA5 and MERRA2 (Modern-Era Retrospective analysis for Research and Applications, Version 2)), the ZTD can be calculated by atmospheric vertical integration using meteorological parameters. Then time interpolation is performed to obtain the ZTD synchronized with the SAR data and resampled to the pixel position of the unwrapped phase of the interferograms for point-by-point correction.

The refractivity *N* can be expressed as follows [18,55]:

(6) $$N=k_{1}\times \frac{P}{T}+k_{2}\times \frac{e}{T}+k_{3}\times \frac{e}{T}+k_{4}\times W_{cl}+k_{5}\times \frac{ne}{f^{2}}$$

where P is the partial pressure of dry air in Pa, e is the partial pressure of water vapor in Pa, T is the temperature in Kelvin, $W_{cl}$ is the cloud water content in $kg/m^{3}$, ne is the electron density in the ionosphere, and f is the electromagnetic wave frequency. The constant $k_{1}=0.776\;{\mathrm{KPa}}^{-1}$, $k_{2}=0.716\;{\mathrm{KPa}}^{-1}$, $k_{3}=3.75\;\times \;10^{3}\;K^{2}{\mathrm{Pa}}^{-1}$, $k_{4}=1.45\;\times \;10^{3}\;m^{3}{\mathrm{kg}}^{-1}$, and $k_{5}=-4.03\;\times \;10^{7}\;s^{-2}m^{3}$.

The tropospheric delay distance *L* can be obtained by integrating over *N* from the surface ($h_{LS}$) to the top of the atmosphere ($h_{top}$). This paper mainly discussed the influence of tropospheric delays on C-band radar, so there is no further discussion on the influence of delay caused by cloud liquid water content and ionosphere (items 4 and 5 in Equation (6)). We use the equation of state of the density of moist air to simplify to obtain the following formula [53]:

(7) $$L=10^{-6}\left[\frac{k_{1}R_{d}}{{\;g}_{m}}P(h)+\int_{h_{LS}}^{h_{top}}\left(\left(k_{2}-\frac{R_{d}}{R_{v}}k_{1}\right)\times \frac{e}{T}+k_{3}\times \frac{e}{T^{2}}\right)dh\right]$$

where $R_{d}$ is the specific gas constant of dry air ($R_{d}=287.05\;J/\mathrm{kg}/K$) and $R_{v}$ is the specific gas constant of water vapor ($R_{v}=461.495\;J/\mathrm{kg}/K$). P(h) is the surface pressure, $k_{2}-\frac{R_{d}}{R_{v}}k_{1}$ is usually named as $k_{2}^{\prime }=0.233\;{\mathrm{KPa}}^{-1}$. $g_{m}$ is the tropospheric stratified mean gravitational acceleration [18].

The first term of Equation (7) is the ZHD, while the subsequent integral term refers to the ZWD. It is obvious that the ZWD depends in part on the e [56], which needs to be converted from the relative humidity $R_{e}$ to e by the Clapeyron–Clausius equation:

(8) $$e(h){=e}^{*}(h)\frac{R_{e}(h)}{100}$$

(9) $$e^{*}(h)=\left\{\begin{array}{cc} e_{w}^{*}(h)=a_{1}e^{a_{3w}\frac{T(h){-T}_{0}}{T(h){-a}_{4w}},} & T(h)\geq T_{0}\\ e_{i}^{*}(h)=a_{1}e^{a_{3i}\frac{T(h){-T}_{0}}{T(h){-a}_{4i}},} & T(h)\leq T_{i}\\ e_{i}^{*}(h)+\left(e_{w}^{*}(h){-e}_{i}^{*}(h)\right)\frac{T(h){-T}_{i}^{2}}{T_{0}{-T}_{i}}, & T_{0}{<T(h)<T}_{i} \end{array}\right.$$

where $e^{*}(h)$ is the saturated water vapor partial pressure, $e_{i}^{*}(h)$ is the supersaturated ice partial pressure, and $e_{w}^{*}(h)$ is the supersaturated liquid water partial pressure; the constants are $T_{0}=273.16\;K$, $T_{i}=250.16\;K$, $a_{1}=611.21\;\mathrm{hPa}$, $a_{3w}=17.502$, $a_{4w}=32.19\;K$, $a_{3i}=22.587$, $a_{4i}=-0.7\;K$.

The calculated ZTD can be spatially interpolated to remove the tropospheric phase delays from the unwrapped phases according to Equations (3)–(5). The advantages of such methods are the large spatial coverage area. The data sources are widely available and easily accessible. Additionally, the data consider the real atmospheric properties (temperature, humidity, and pressure). Limitations are that the spatial resolution is lower than that of SAR data, so the small-area delay correction is not significant. Its time may differ from SAR data by several hours.

#### 2.2.4. Methodology Based on PWV

PWV is defined as the column height of liquid water formed by condensation of water vapor, which corresponds to the total water vapor content from the earth’s surface to the top of the atmosphere [57]:

(10) $$PWV=\frac{1}{\rho_{w}R_{v}}\int_{h_{LS}}^{h_{top}}\frac{h}{t}dh$$

where $\rho_{w}$ is the density of liquid water ($\rho_{w}=1.0\;\times \;10^{3}\;\mathrm{kg}/m^{3}$). The InSAR interferograms tropospheric wet delayed phases $\phi_{tropo}^{wet}$ can be converted from Equation (11):

(11) $$\phi_{tropo\;}^{wet\;}=-\frac{4\pi }{\lambda }\frac{\Pi }{cos(\theta )}PWV$$

where Π is a dimensionless conversion factor that varies with the troposphere’s temperature [41,57,58]. If the tropospheric weighted average temperature $T_{m}$ is known, the conversion factor can be obtained from Equation (12):

(12) $$\Pi =0.102+\frac{1708.08\left[K\right]}{T_{m}}$$

where $T_{m}$ can be calculated by the GPT3 model [59], a linear regression model based on surface temperature [57], or the GTm model [60].

Based on the PWV data, only ZWD can be obtained. The ZHD variation with respect to the long-time baseline will not be effectively removed, which can be estimated using the Saastamoinen model or combined with the ZWD using the ZHD calculated by other products to obtain the ZTD. Spatial interpolation can then be performed according to Equations (3)–(5) to remove the tropospheric phase delays from the unwrapped interferogram phase.

The advantage of such methods is the high spatial resolution, generally reaching 300~1000 m (OLCI, MODIS), which other data cannot achieve. The data are actual independent measurements. The limitation is that the time difference between data acquisition and SAR data is large, resulting in the inability to obtain the real atmospheric state data at the time of SAR data acquisition. Additionally, it is easily affected by cloud cover, resulting in the complete unavailability of data at individual moments. Inaccurate cloud masks will have an impact on data reliability.

#### 2.2.5. Methodology Based on the Interferogram Phases

There are three main correction methods based on the interferogram phases. The first is the interferogram stacking method, which uses the principle of phase stacking to improve the signal-to-noise ratio of the interferograms; the second is an empirical method based on a single interferogram, which constructs a functional relationship between the unwrapped phase and the topography estimate the topography-dependent delay signals. Empirical methods are represented by two models that consider a linear correlation between the tropospheric delays and the surface elevation and another that thinks a power–law relationship between them [22,61,62,63]; the third one is a time-series-based spatiotemporal filtering approach, in which a stable regional window close to the deformation region is selected. The atmospheric delay signals of the window are extracted by quantitative self-covariance analysis. Then the atmospheric delay signals of the deformation region are estimated using the covariance function correlation property.

These methods are simple, fast, and unaffected by external data defects. However, the interferogram stacking method and spatiotemporal filtering method do not consider the non-Gaussian distribution of tropospheric delays, which will reduce the temporal resolution and lose the important surface displacement signals. Moreover, spatiotemporal filtering is only effective for the turbulent mixed delays. Since the empirical methods seek the relationship between interferogram phases and topography, they have good correction effect for the interferogram where the vertical stratification delays component is dominant. However, when the turbulent delay component is dominant or the surface deformation is elevation dependent, such methods cannot separate the tropospheric delays and the surface deformation signals well.

## 3. Data Processing

### 3.1. InSAR Data Processing

**Figure 3:**
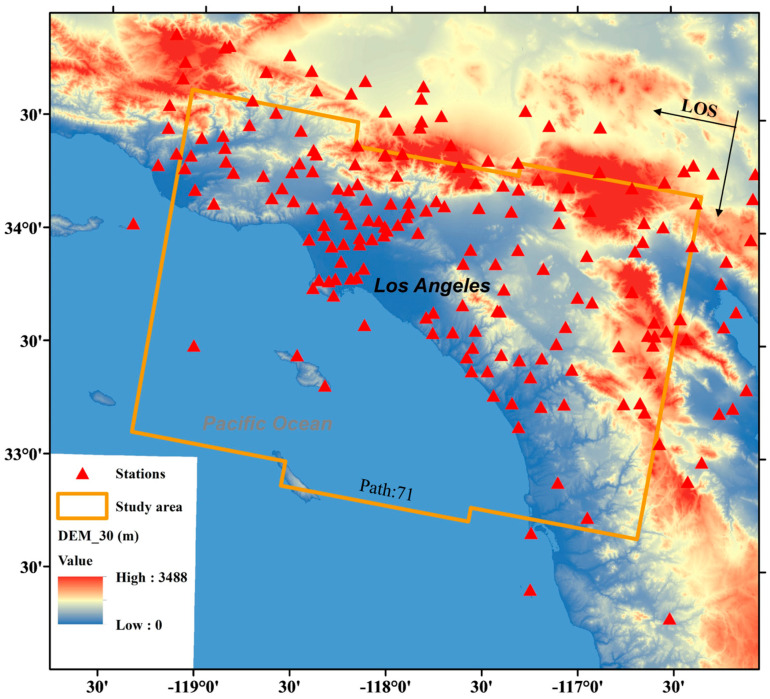
Study area and the distribution of GNSS stations. The orange border indicates the data boundary of frame 480 of Sentinel-1 orbit 71, which is also the experimental area of this study. The bottom panel shows the STRM 30m resolution DEM data. Red triangle symbols indicate GNSS stations inside and around the study area. The station selection boundary was expanded by 20 km to ensure the accuracy of the data at the boundary of the study area.

**Figure 4:**
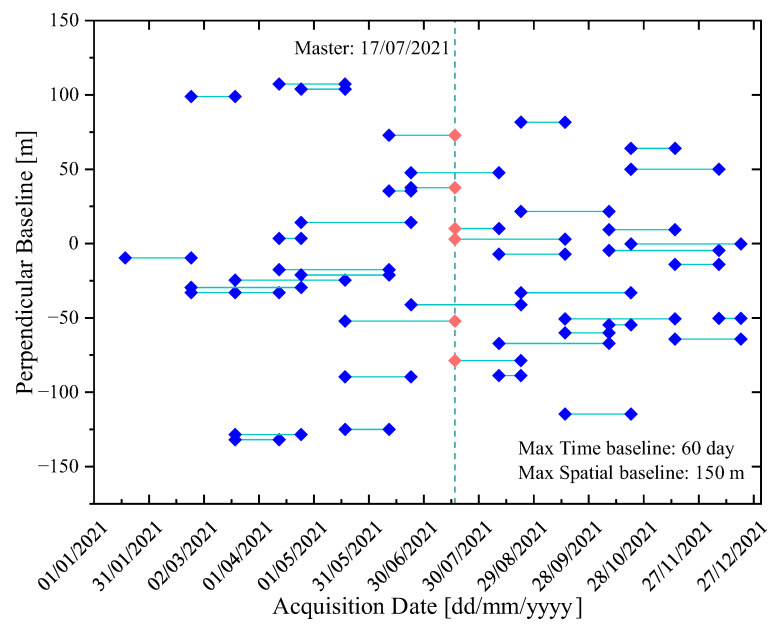
Small baseline interferogram. The blue diamond indicates the slave images under each date and red diamond indicates the master image selected on 17 July 2021. The green line indicates the small baseline interferograms for each image.

This paper used the C-band Sentinel-1 descending-orbit data (Path:71) at 12 or 24-day intervals from 18 January 2021 to 20 December 2021, a total of 17 single-look complex (SLC) data. The data acquisition time is 13:52 (UTC). With a land coverage of approximately 29,000 km^2^, the study area is shown in Figure 3:

In this paper, all SLC data were preprocessed using the stack processing of the ISCE (Interferometric synthetic aperture radar Scientific Computing Environment) [64,65]. We selected the SLC data on 17 July 2021 as the reference to provide the reference geometry. Other SLC data were coregistered and resampled to this reference geometry using the 30 m resolution DEM and precise orbit ephemerides. The maximum temporal and spatial baseline thresholds were set to 60 days and 150 m to ensure a strong spatiotemporal correlation, and 42 interferograms were obtained under this spatiotemporal baseline parameter. Figure 4 shows this small baseline map.

The generated 42 differential interferograms are imported into StaMPS (StaMPS 4.1-beta) for SBAS reprocessing. In order to reduce the difficulty of subsequent data calculation, a multi-look operation with 20 pixels in the azimuth direction and 5 pixels in the range direction was performed. Then, we selected the pixel points with coherence greater than 0.6 for 2D phase unwrapping. Ten iterative cycles were performed in this step to ensure the unwrapping effect of the data.

### 3.2. Tropospheric Delays Data Processing

#### 3.2.1. GACOS

GACOS is a widely used InSAR tropospheric delay correction product [47,54]. The iterative tropospheric decomposition model separates the vertically stratified and turbulent mixing delays from the tropospheric delays and reconstructs a high spatial resolution ZTD map to correct InSAR tropospheric delays and other applications. The temporal resolution is 1 min and the spatial resolution is 90 m.

We first preprocessed the acquired GACOS ZTD products using the TRAIN (Toolbox for Reducing Atmospheric InSAR Noise) [66], generating the corresponding differential delays from the small baseline list. Then, we projected the differential ZTD to the LOS direction and resampled the data to interferometric pixel positions.

#### 3.2.2. BP + GNSS

**Figure 5:**
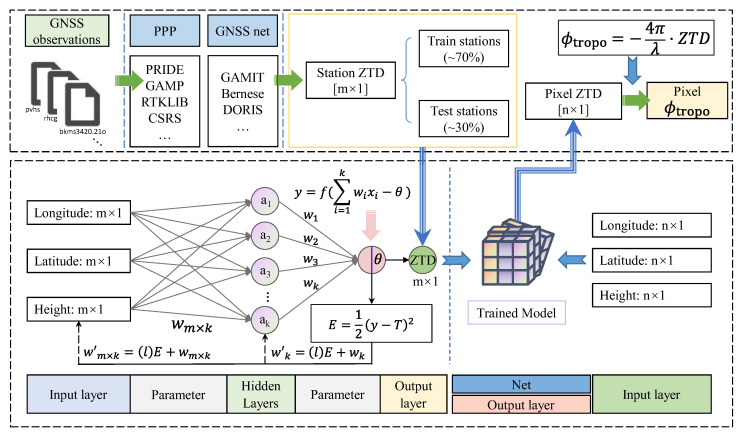
The framework of the BP + GNSS method. The upper dashed box is the main process part. The lower dashed box is part of GNSS ZTD spatial prediction by the BP neural network.

Since the ZTD data obtained by GNSS were spatially discrete, it was necessary to interpolate the GNSS ZTD to the interferometric pixel positions to be able to correspond to the interferometric pixels. However, the traditional spatial interpolation method only considers the spatial features of the data without considering the actual geographical features. For this reason, this paper proposed a spatial prediction method of GNSS ZTD based on the BP neural network. The main technical process is shown in Figure 5.

The main process was as follows:

Step 1: GNSS stations selection

The date of SAR data acquisition was used as a reference to select GNSS ground stations in the study area. In order to ensure the accuracy of the interpolation at the boundary of the study area, the range of selected ground stations was extended by 20 km, with about 200 valid stations per day.

Step 2: Solving GNSS to obtain ZTD

To obtain the ZTD measured by GNSS ground stations, we grouped the observation files from GNSS stations and solved the baseline using GAMIT. Four IGS stations, FAIR (147.50° W, 64.97° N) in Alaska, North America, BOGT (74.08° W, 4.64° N) in Bogota, Colombia, WARK (46.05° W, 60.72° N) in southern Greenland, and QAQ1 (174.05° E, 60.72° S) in North Island, New Zealand, were added to each group to improve the accuracy of the solution. The GNSS ZTD data with a temporal resolution of 5 min were extracted after solving three sets of GNSS data under each date.

Step 3: Prediction of interferometric pixel position ZTD

Firstly, we trained the BP neural network using GNSS ZTD and elevation data. Specifically, 70% of the sites were selected as the training set for model training, which was used for the gradient descent of the model; 15% of the sites were used as the verification set, which were used to verify the accuracy of the model after each epoch was completed; 15% of the sites were used as the test set, which were used for testing the accuracy of the trained model. The model was considered to have achieved the training effect when the R-values of the training set, validation set, and test set were all greater than 0.99. Then the latitude, longitude, and elevation data of the pixel points of the interferogram were input to the trained model. Finally, the ZTD of the pixel positions of the interferogram predicted by the BP neural network was obtained at the end of the model run.

Step 4: Phase delays calculation in the LOS direction

We employed the model predicted ZTD values to obtain the STD in the LOS direction by Equation (3) and converted the STD into phase delays by Equation (4).

**Figure 6:**
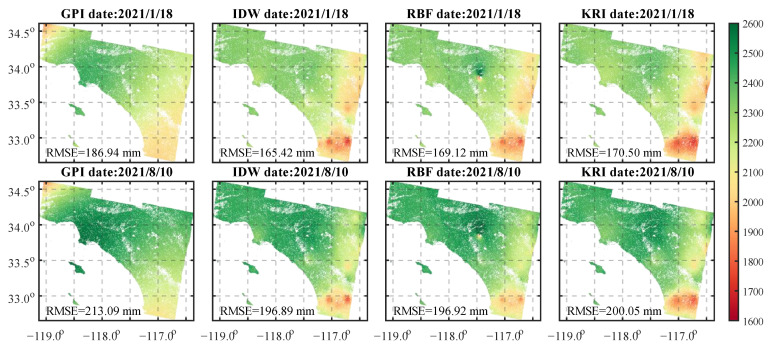
Comparison of interferometric pixel position ZTD interpolation based on GPI, IDW, RBF, and KRI methods for two days, on 18 January 2021 and 10 August 2021.

We compared the interpolation effect of GPI, IDW, RBF, and KRI methods in this region (Figure 6). The results showed that the RMSEs of both the deterministic method of GPI, IDW, and RBF and the geostatistical method of KRI were larger, with an increase in RMSE of 16.61% from winter to summer. The difference between the interpolated ZTD and the original ZTD relates to the station elevation. The interpolated ZTD of the region with high water vapor content at low altitude was too large and the difference gradually decreased with the increase in altitude. When the altitude was higher than 2000 m, the interpolated ZTD was gradually too small, and the difference became larger and larger as the altitude continued to increase. The reason was that the water vapor content at different heights was different, resulting in changes in the delay signal, which indicated that this method was unable to effectively capture the water vapor differences caused by the complex terrain within the region. Therefore, it is not suitable for spatial interpolation of the phase delays in areas with significant terrain changes.

**Figure 7:**
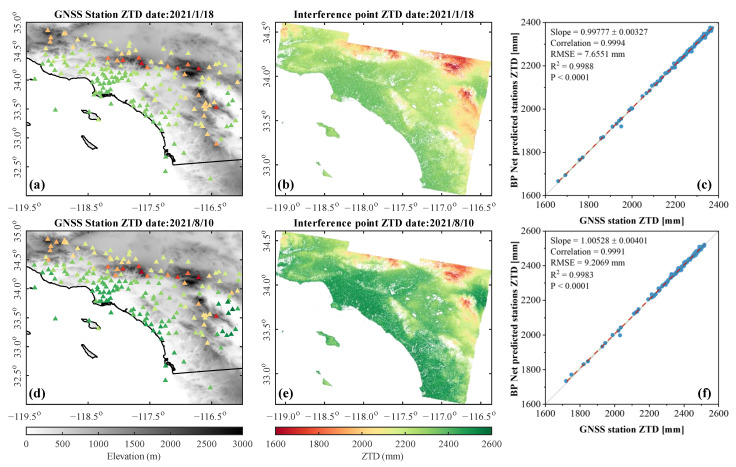
Comparison of BP predicted ZTD and GNSS ZTD. ZTD distribution in the study area on 18 January 2021 (first row) and 10 August 2021 (second row). (**a**,**d**) are GNSS site ZTD data solved using GAMIT. The gray bottom panel is DEM data. (**b**,**e**) are ZTD data on spatially predicted interferometric pixel locations using BP Net. (**c,f**) are correlation analysis of the BP Net forecasted GNSS site ZTD and the original GNSS site ZTD. The blue circle is the ZTD value, the gray straight line is the Y = X reference line, and the red dotted line is the linear fit of the scatter points..

The ZTD obtained based on the BP + GNSS spatial prediction method was statistically validated in this paper. The ZTD at GNSS sites was extracted from the predicted ZTD maps and compared with the solved ZTD at each GNSS site. Figure 7c,f show the correlation analysis between the predicted ZTD based on the BP + GNSS method and the GNSS ZTD at GNSS sites on 18 January 2021, and 10 August 2021. The RMSE for the two days were 7.66 mm and 9.21 mm, respectively. The correlations were greater than 99.9%, and the *p*-values were less than 0.0001, which proved the effectiveness of the BP + GNSS method. In Table 1, we provided the R^2^ of the model input and output quantities for all dates, and the RMSE of the model output ZTD and the original GNSS ZTD for each date. RMSE was reduced by about 95.5% compared with conventional interpolation methods.

#### 3.2.3. ERA5

ERA5 is the latest global atmospheric reanalysis data with a temporal resolution of 1 h and a spatial resolution of 0.25°. The calculation of the ERA5 ZTD requires the acquisition of vertically stratified meteorological data: pressure, temperature, and relative humidity, with 37 vertical layers for each data type, corresponding to pressure layers of 1000 hPa to 1 hPa.

In this paper, the ERA5 stratified data on the day of SAR data were obtained from CDS (Climate Data Store). Since the ERA5 data were not synchronized with the SAR data in the study area in time (the SAR data acquisition moment was 13:52, and the vicinity of time were 13:00 and 14:00), we used Equations (6)–(9) to calculate the ZTD value of ERA5 at 13:00 and 14:00. We then interpolated it to the SAR acquisition time. Finally, we converted them to the LOS direction tropospheric phase delays using Equations (3) and (4), and removed them from the unwrapped phase using Equation (5).

#### 3.2.4. MERRA2

MERRA2 data are atmospheric reanalysis data provided by NASA. The horizontal resolution is 0.5° × 0.625°, the vertical resolution is 42 pressure levels up to 0.1 hPa, and the temporal resolution of the pressure level data is three hours.

This experiment acquired MERRA2 data from NASA’s GES DISC (Goddard Earth Sciences Data and Information Services Center). The data processing process uses the TRAIN. The tropospheric phase delays at each baseline interferometric pixel position could be obtained by scripting.

#### 3.2.5. MODIS PWV

The MODIS sensor can acquire global atmospheric water vapor data with a revisit period of 1–2 days and a spatial resolution of 1 km. The high resolution of the data can effectively support the high sensitivity of the interferograms to water vapor differences in small areas. Therefore, MODIS PWV data are also included as a data source for interferogram tropospheric phase delay correction, and its correction effect is analyzed in this paper.

The MODIS PWV with the same date as the SAR were obtained from NASA’s DAAC (Distribution System Distributed Active Archive Center). Since the infrared and near-infrared bands of MODIS cannot penetrate the clouds, it leads to a large error in the water vapor inversion in the cloud-covered region. Before using MODIS data for InSAR tropospheric phase delay correction, this paper combined the MODIS cloud mask data (MOD35) to remove the data disturbed by clouds pixel by pixel and retained only the data in clear sky conditions. Then, we filled the blank pixels in the study area by interpolation. Finally, PWV was converted to LOS direction tropospheric phase delays $\phi_{tropo}^{wet}$ by Equations (10)–(12), and then combined with ERA5 $\phi_{tropo}^{hydr}$ to form the $\phi_{tropo}^{}$.

#### 3.2.6. Ph-Elev (Linear)

The linear correction method based on the interferogram phases and topography significantly affects the vertical stratification delay component but not the turbulent mixed delay component. The linear correction was included in this paper for comparison and combined with the analysis of other correction methods. Since the study area was selected with significant topographic variation (0~3000 m), it is possible to observe the correction performance of linear correction in small regions with significant topographic variation. The linear correction can determine the dominance of vertical stratification delay and turbulent mixed delays in the phase delays of each interferogram to observe the correction effect of other methods when linear correction is not significant (turbulent mixing delays component dominates). This will support combining the advantages of empirical and external data-based correction methods [67]. The TRAIN calculated the phase delays based on Ph-Elev (Linear) corrections, where the topographic data were obtained from STRM 30 m resolution DEM data.

## 4. Tropospheric Delays Correction Result of the Unwrapped Phases

### 4.1. Statistical Methods for Assessing the Quality of Corrections

In this paper, the calibration quality was evaluated by StaD and SSIM to verify the correction effect of each method. StaD can well reflect the dispersion of the unwrapped phases after calibration by various methods. SSIM’s main feature is that it can well reflect the spatial structure difference of the unwrapped phases before and after correction. The calculation is shown in Equations (13) and (14):

(13) $$StaD=\sqrt{\frac{1}{M-1}\sum_{i=1}^{M}\left(\phi_{{corr}_{{int\;}_{i}}}-\bar{\phi_{{corr}_{int\;}}}\right)}$$

where $\phi_{{corr}_{{int\;}_{i}}}(i=1,2,3,\ldots ,M)$ is the corrected M interferogram unwrapped phases, and $\bar{\phi_{{corr}_{int\;}}}$ is the average of the M corrected phases. If no significant surface displacement occurs within the temporal baseline, there is essentially no significant streak information on the interferograms. Hence, the unwrapped data should have no obvious dispersion, and StaD should be smaller.

(14) $$SSIM\left(\phi_{int\;},\phi_{{corr}_{\;int}}\right)=\frac{\left(2\mu_{\phi_{int\;}}\mu_{\phi_{{corr}_{\;int}}}+C_{1}\right)\left(\sigma_{\phi_{int\;}\phi_{{corr}_{\;int\;}}}+C_{2}\right)}{\left(\mu_{\phi_{int\;}}^{2}+\mu_{\phi_{{corr\;}_{int}}}^{2}+C_{1}\right)\left(\sigma_{\phi_{int\;}}^{2}+\sigma_{\phi_{{corr}_{\;int\;}}}^{2}+C_{2}\right)}$$

where $\mu_{\phi_{int\;}}$ and $\phi_{{corr}_{\;int}}$ refer to the mean value of the unwrapped phases before and after correction and $\sigma_{\phi_{int\;}}^{}$ and $\sigma_{\phi_{{corr}_{\;int\;}}}^{}$ are the standard deviation of the unwrapped phases before and after correction, respectively. $\sigma_{\phi_{int\;}\phi_{{corr}_{\;int\;}}}$ is the covariance of the unwrapped phases before and after correction. To avoid the case that the denominator is zero, $C_{1}$, $C_{2}$ constants are introduced, $C_{1}={\left(k_{1}\times L\right)}^{2}$, $C_{2}={\left(k_{2}\times L\right)}^{2}$, $k_{1}=0.01$, $k_{2}=0.03$; L is the dynamic range of the input image. The larger the value, the smaller the phase difference between the interferogram unwrapped before and after correction for different methods of the same interferogram, i.e., the lower the degree of correction. Conversely, the smaller the SSIM data, the more adequate the correction is. If it is significantly smaller than the other methods, it may be overcorrected.

### 4.2. Analysis of Correction Results

This paper calculated the tropospheric phase delays of 42 interferograms using five commonly used tropospheric delay correction methods and the BP + GNSS method, respectively. They were used to correct the unwrapped phases of interferograms. Table 2 statistics the mean of StaD, SDP (StaD decrease percentage), and SSIM values before and after correction for all interferograms, NISD (number of interferograms for StaD decrease) and CPIN (correction percentage of interferogram number) are also given. Ph-Elev (linear) was the only method in which the StaD of all 42 interferograms decreased after correction. This means that all interferograms include the vertically stratified delays and are suppressed. ERA5 has a higher spatial resolution than MERRA2, but the mean value of StaD after correction was higher than that of MERRA2, and the SSIM of ERA5 was slightly lower than that of MERRA2. It showed that the phase delays calculated using the ERA5 method were slightly higher than those of MERRA2. The CPIN of MODIS is higher than that of ERA5, but the mean of StaD after correction was the largest. The CPIN of GACOS was lower than Ph-Elev (linear) method, but the mean of StaD decrease percentage is higher than the Ph-Elev (linear). The CPIN of BP + GNSS was slightly lower than Ph-Elev (linear), but the average StaD decrease percentage was significantly higher among other methods (52.03%).

**Figure 8:**
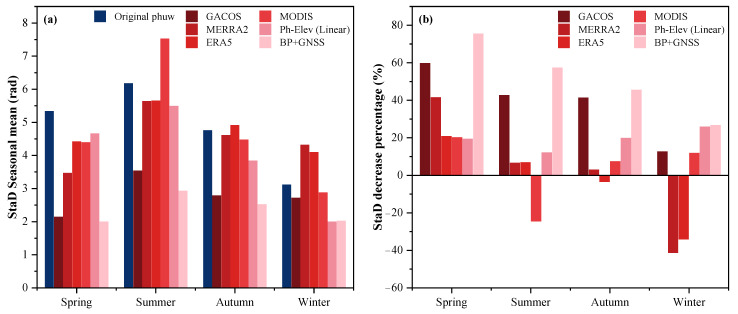
The StaD of the uncorrected interferometric phases for each season (the leftmost dark blue bar in each group). The mean value of the StaD of the interferograms for each season after correction by various correction methods is shown in the figure. The methods corresponding to bars 2–7 in (**a**) and 1–6 in (**b**) are GACOS, MERRA2, ERA5, MODIS, Ph-Elev (linear), and BP + GNSS, respectively. And correspond to the respective legend colors.

Figure 8 is a comparison of the correction performance of several correction methods in each season. Figure 8a shows that the mean value of the StaD of the interferograms before correction is the largest in summer and the smallest in winter. Since the southwest of downtown Los Angeles is the Pacific Ocean, the wind field from southwest to northeast in summer brings a large amount of water vapor over the Pacific Ocean, significantly impacting the propagation of radar signals in the atmosphere. In contrast, the water vapor content is lower in winter, which interferes less with the propagation of radar signals. ERA5 in autumn and winter and MERRA2 in winter both show that the corrected StaD is larger than the original interferograms, i.e., the correction rate after the correction has a negative value (Figure 8b). This is because the spatial resolution of ERA5 and MERRA2 are significantly low. The water vapor content is relatively reduced in autumn and winter, which amplifies the disadvantage of the low spatial resolution of the data and cannot reflect the small regional differences of water vapor in the coastal region, thus leading to the poor quality of the correction. The quality of the interferograms deteriorates after correction by MODIS in summer because the water vapor content in summer is large, and the changes are complex. The time difference between the MODIS data and the SAR data in the Los Angeles area is about 6 h (Figure 2b), which will lead to a large difference between the MODIS water vapor after cloud removal and the water vapor in the SAR data, making the phase delay’s correction either insufficient or overcorrected. The seasonal correction effect of the InSAR tropospheric phase delays calculated by GACOS and the proposed BP + GNSS method was also compared. The StaD of the GAOCS product are 2.15, 3.54, 2.79, and 2.72 rad for each season, respectively. The StaD of the BP + GNSS correction was 1.99, 2.93, 2.52, and 2.02 rad for each season, respectively. The StaD of the corrected unwrapped phases of the BP + GNSS method was better in summer and winter, and the two are comparable in spring and autumn. This demonstrates that the BP + GNSS method can accurately remove tropospheric phase delays not only in conditions of low water vapor content but also maintains excellent correction performance in situations of higher water vapor content, exhibiting the capability to overcome variations in water vapor due to complex terrain.

**Figure 9:**
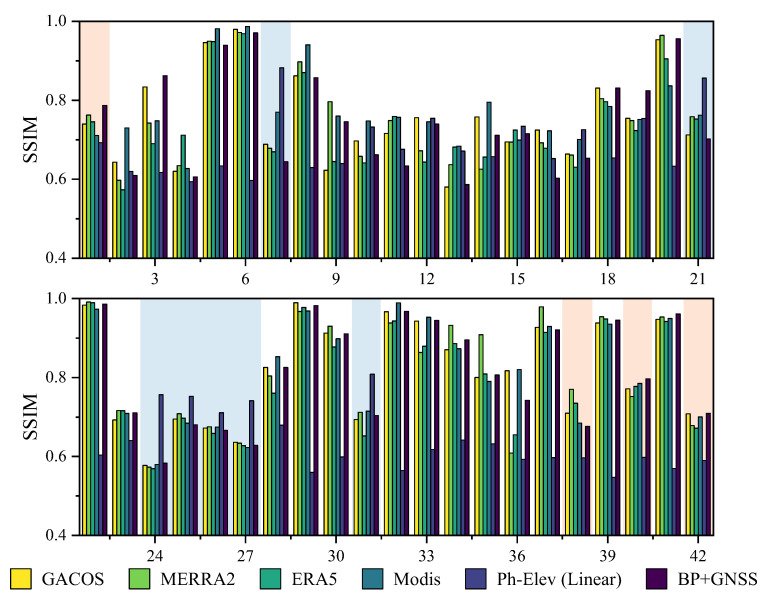
Comparison of SSIM indices of the original interferograms unwrapped phases corrected by each method. The first row shows the first 21 interferograms, the second row shows the last 21 interferograms, and each group of 6 bars corresponds to the 6 correction methods in the lower legend. The interferograms with orange backgrounds are best corrected by the Ph-Elev (linear) method. The blue background indicates the dominance of turbulence in the phase delays of this interferogram.

Figure 9 shows that the method based on Ph-Elev (linear) has lower SSIM values in all interferograms, which implies that such methods can effectively remove the vertically stratified delays due to terrain height variation. We suppose the Ph-Elev (linear) method can still correct about 60% of the delayed structure when the tropospheric phase delay’s structure accounts for about 20–30%. In that case, this method is better than the GACOS and BP + GNSS methods (orange background fill in Figure 9), and this phenomenon is concentrated in winter. This is because the phase delays of the vertical stratification dominate when the atmospheric water vapor content is low and the atmospheric turbulence is weak in winter. The Ph-Elev (linear) method is good at removing the delays caused by a small increase in water vapor content. The proportion of tropospheric phase delay in some interferograms was less than 10%. However, the Ph-Elev (linear) method still maintains a high correction performance (SSIM~0.6), and the StaD of the Ph-Elev (linear) method was much higher than that of the GACOS and BP + GNSS methods at this time. This phenomenon indicates that the Ph-Elev (Linear) method may be overcorrected during periods of low water vapor content. When the SSIM of the Ph-Elev (linear) method is significantly higher than the other methods, it is considered that the turbulent delays dominate at this time, and the higher part is the remaining turbulent delays after the correction of the Ph-Elev (linear) method.

**Figure 10:**
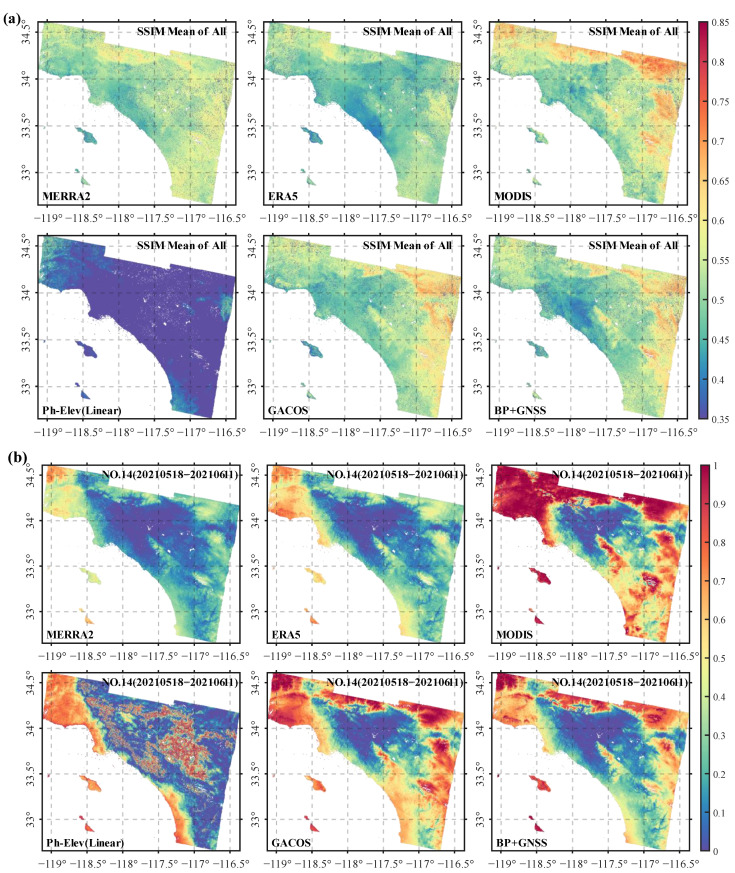
Spatial distribution of SSIM before and after interferogram correction. (**a**) is the spatial distribution of the average SSIM of 42 interferograms before and after correction by each method and (**b**) is the SSIM spatial distribution of a pair of interferograms (18/05/2021–11/06/2021) with a longer perpendicular baseline.

Figure 10 displayed the spatial average of SSIM for 42 interferograms after correction by various methods (Figure 10a) and the corrected SSIM of an interferogram with a longer perpendicular baseline (Figure 10b). The average SSIM results for the 42 interferograms after correction showed no significant spatial differences by the MERRA2 and ERA5 methods, inconsistent with the noticeable terrain differences within the region. However, the MODIS method exhibited the most significant spatial differences, particularly showing larger SSIM values in areas with pronounced terrain changes and high elevations. Analyzing the correction effects of MERRA2, ERA5, and MODIS, it was believed that these differences were primarily due to the spatial resolution of the data itself. MERRA2 has the lowest spatial resolution, resulting in minimal spatial differences. ERA5 has a slightly higher resolution than MERRA2, exhibited better correction effects in urban areas than MERRA2. Still, the effects were essentially similar in areas with noticeable terrain changes in the east and north. This indicated that data with lower spatial resolutions are not suitable for correcting InSAR atmospheric delays in regions with evident terrain changes located along coastal areas. Despite the high resolution of MODIS data, the overestimation of atmospheric delay and the impact of cloud cover lead to non-smooth spatial occurrences, especially in urban and mountainous transition areas and high-altitude regions (Figure 10b). This indicated that the MODIS method was subject to numerous external limiting conditions, primarily the temporal differences with SAR data and susceptibility to cloud interference, resulting in a loss of correction performance during active atmospheric water vapor periods. The Ph-Elev (linear) method exhibits apparent overcorrection. The SSIM values corrected by this method were notably smaller than those of several other methods and did not exhibit apparent spatial variations. Figure 10b displayed noise in transitional areas between urban and mountainous regions when using this method. The GACOS and BP + GNSS methods showed spatial differences that aligned with terrain features, achieving better results because both methods are based on GNSS ZTD for correction. However, in the eastern and northern mountainous regions, the SSIM of the BP + GNSS method was relatively lower compared to the GACOS method, proving the superiority of our proposed method in areas with pronounced terrain changes. We attribute this improvement to the introduction of the BP neural network, which better captures the variations in atmospheric delay with terrain changes, resulting in more accurate predictions of spatial water vapor values and ultimately achieving better and more stable correction effects.

**Figure 11:**
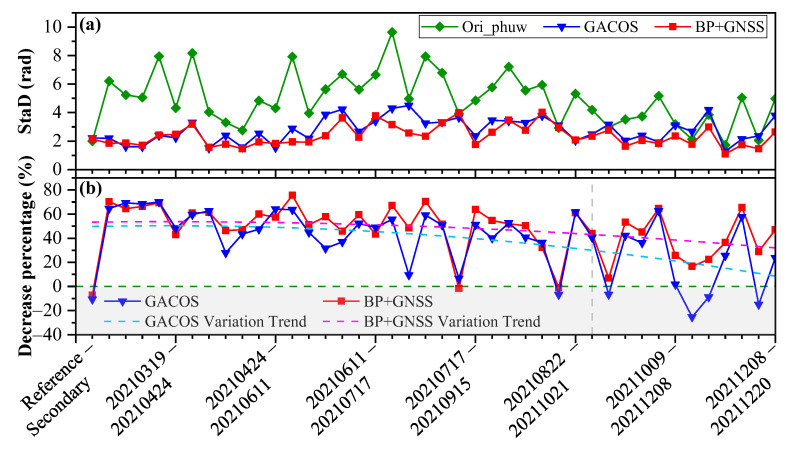
The StaD of 42 interferograms before and after correction based on CACOS and BP + GNSS method (**a**) and correction rate of two methods (**b**). The gray vertical dashed line is the interferogram 15/09/2021–09/10/2021, the green horizontal dashed line represents the correction effect zero point, and the lower gray filled area represents the worse effect after correction; the other two dashed lines are the quadratic polynomial fit to the data to show the trend of the effect of the two methods.

Figure 11a compares the SatD of 42 interferograms before and after correction by GACOS and BP + GNSS methods. The results showed that 85.71% of the StaD corrected by the BP + GNSS method were lower than the GACOS method, and the corresponding StaD is 14.00% lower than the GACOS method on average. Overall, the StaD fluctuation of the time-series small baseline interferograms corrected by the BP + GNSS method was smaller than that of the GACOS method, especially in the spring when the water vapor changes were not obvious. Figure 11b compares the StaD decrease percentage after correction between the GACOS and BP + GNSS methods. The StaD of 14.29% of the interferograms increased after correction by the GACOS method, while the StaD of 7.14% of the interferograms increased by the BP + GNSS method. From the two StaD downward trend lines, we found that the downward trend of the GACOS method accelerated after the interferogram 15/09/2021–09/10/2021. The StaD downward trend line of the BP + GNSS method changed significantly slower than that of the GACOS method, indicating that the BP + GNSS method has stable performance in the time series InSAR tropospheric phase delay correction.

**Figure 12:**
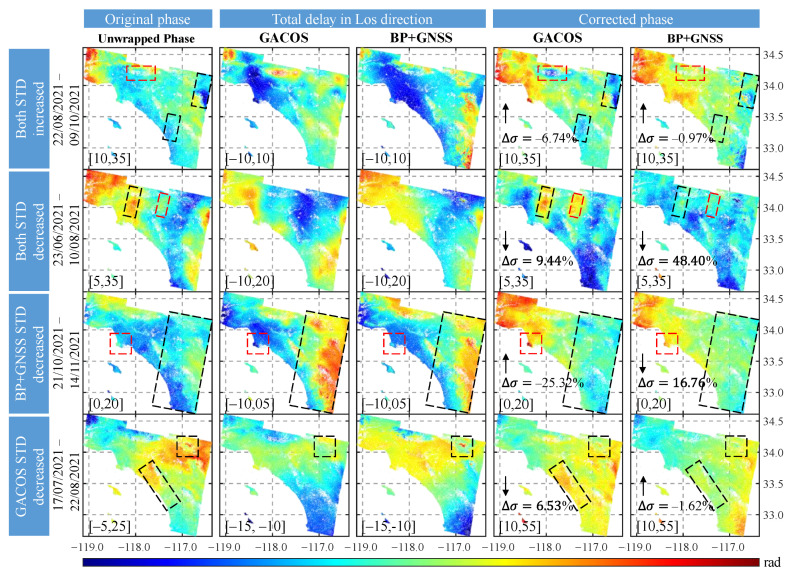
Comparison of BP + GNSS and GACOS correction effects. The first column shows the original interferogram unwrapped phases; the second and third columns show the total phase delays (LOS direction) in the troposphere under the two methods; the last two columns show the corrected interferogram unwrapped phases. Where the arrows indicate the trend of the StaD change (increase/decrease) after correction, and ∆σ is the percentage of StaD change; the red box indicates that the region brings in new deviation after correction, and the black box indicates that the region has no obvious effect after GACOS correction. All subplots share a color band, and the value in the lower left corner of each subplot in parentheses is the mapping interval of the color band value for that subplot.

Figure 12 compares the four cases of StaD change in the interferograms after the correction based on GACOS and BP + GNSS methods, respectively. When the StaD increased after correction (first row, interferogram: 22/08/2021–09/10/2021), the StaD increased by 6.47% after correction based on the GACOS method. Additionally, compared to the original interferogram, the method introduced new deviations in the area marked by the red box in the study area and did not make good ease of the deviations in the two black boxes. After comparing with the topography, we found that the red box was located in the mountainous area near the urban area with large topographic differences (500~3000 m), which might lead to new errors. Still, this area was improved by the BP + GNSS method. When the corrected StaD were all reduced (second row, interferogram: 23/06/2021–10/08/2021), the GACOS method reduced the StaD by 9.44% but introduced new deviations (red boxed area). The BP + GNSS method reduced it by 48.4%. The BP + GNSS method was more effective than the GACOS method in correcting the deviations in the black boxed area. When the GACOS StaD increased after correction, but the BP + GNSS method decreased (third row, interferogram: 21/10/2021–14/11/2021), new deviations appeared in the Rolling Hills (red dashed box) southwest of Los Angeles after correction with the GACOS method, where the topographic variation is larger (0~300 m) than in the surrounding area. No anomalies appeared after correction based on the BP + GNSS method, and the results were better after correction on the east side (black dashed box). When the GACOS StaD decreased after correction, but the BP + GNSS method increased (fourth row, interferogram: 17/07/2021–22/08/2021), the StaD decreased by 6.35% after GACOS correction and increased by 1.62% after BP + GNSS method correction. However, the BP + GNSS method corrects in the black box (higher elevation) outperformed the GACOS method.

## 5. Discussion

The tropospheric water vapor is affected by seasonal, topographic, and climatic conditions as well as other factors and presents the characteristics of large variability in spatial distribution and rapid spatiotemporal changes. Meanwhile, SAR data acquisition time is fixed at a certain moment (Figure 2). Therefore, the data used for InSAR tropospheric phase delays correction need to be similar to SAR data in time, i.e., data with high temporal resolution and good continuity are more advantageous for correction, such as GACOS products and GNSS ZTD data, and the StaD after correction decreases by 44.04% and 52.03% on average, respectively (Table 2).

When MODIS PWV data is used for InSAR tropospheric delay phase correction, it is necessary to consider that MODIS data is easily disturbed by clouds, and the calculated water vapor value is high. Therefore, the pixels affected by clouds should be cleared and combined with GNSS or radiosonde data for numerical calibration [41]. The temporal difference between MODIS and SAR data should also be considered, as the difference between MODIS and SAR data is usually several hours (Figure 2b), which may lead to a large difference in water vapor between the two moments, especially during the summer when the troposphere is active. The difference between MODIS data and SAR data in the experimental area of this study is about 6 h. In summer, the StaD of the interferograms corrected for phase delays calculated by MODIS PWV increased by 24.56%. This proves that MODIS data with too much time difference from SAR data cannot be directly used for InSAR tropospheric phase delay correction during the active water vapor period.

Due to the high temporal resolution, large coverage area, and easy data acquisition of reanalysis meteorological data, many scholars have tried to use it to correct InSAR tropospheric phase delays [29,45,66]. In this paper, we compared the correction effects of MERRA2 and ERA5 in the study area. The StaD of the unwrapped phase decreased by 41.64%, 6.73%, 3.10%, −41.39%, and 20.86%, 6.94%, −3.41%, and −34.09% (Figure 8) in the four seasons after the correction of both methods. We analyzed the phenomenon that the correction effect was better in spring and not obvious in summer, while some StaD increased after correction in autumn and winter, which is attributed to the small-scale study area (the minimum length of land along the orbital direction is about 60 km, and the minimum linear distance of land in the vertical orbital direction is about 53 km (Figure 3)), and the overall arc-shaped narrow area, while the spatial resolution of ERA5 in the study area is about 25 km, the spatial resolution of MERRA2 is about 47 × 70 km. Therefore, this paper considers that these two data cannot effectively inhibit the small coastal area InSAR tropospheric phase delays. However, the change in tropospheric water vapor in spring was small, so the SatD decrease effect was more obvious after correction.

GACOS achieved a good correction effect in this experimental area, and the average StaD of all the corrected interferograms decreased by 44.04%, which is much higher than the four methods except for the BP + GNSS method. The better correction is attributed to including high temporal resolution ZTD observations from GNSS ground stations and the dense and uniform distribution of GNSS stations in this region.

The BP + GNSS method achieved the best results in this experiment, with 92.86% of the interferograms corrected and an average decrease in the StaD of more than 50% (52.03%). We attribute its good correction results to the following reasons:

In this paper, surface elevation data (Figure 5) were incorporated into the BP + GNSS model training and prediction processes to provide a topographic reference factor for the model. The interpolation process considered the topographic features within the study area, thus capturing the variation pattern of atmospheric features in small spaces between GNSS stations. It also allowed the model to capture the nonlinear relationship between atmospheric feature variation and topographic relief, ensuring accurate interpolation between GNSS stations and continuity of interpolation across the interferograms.

Expanding the station selection range ensured the accuracy of boundary position prediction. Directly predicting the ZTD of the pixel position of the interferogram, avoided additional errors caused by operations such as resampling.

The dense and spatially uniform distribution of GNSS stations in the study area will greatly affect the accuracy and reliability of the GNSS ZTD spatial interpolation. The denser and more uniformly distributed stations will make the interpolated delayed phase map closer to the real atmospheric phase delays.

## 6. Conclusions and Outlook

Tropospheric phase delays have been a major challenge, limiting InSAR in achieving high-accuracy earth observations. It varies with regional location, topography, and atmospheric state, making finding a uniform method for effective suppression difficult. In order to further explore the best means of InSAR tropospheric phase delay correction, this paper proposed a correction method based on GNSS ZTD and BP Network, validated the correction effect by taking Los Angeles in the southwest US region as an example, and compared it with several commonly used InSAR tropospheric phase delay correction methods to draw the following conclusions:

The Ph-Elev (linear) method decreased the StaD in all corrected interferograms but caused new noise signals in the unwrapped phases in areas with significant topographic variations. Additionally, there might be some overcorrection during periods of low water vapor content, indicating that the method may remove the true surface deformation information. The spatial resolution of ERA5 and MERRA2 data is low, and the delayed correction for small-scale areas is not good. The correction performance of GACOS data was degraded in high-altitude areas.

The BP + GNSS method proposed in this paper performed BP neural network spatial predictive interpolation of GNSS sites for the mismatch between the spatial resolution of GNSS data and the pixel points of InSAR interferograms. We found that the method achieved significant results for InSAR tropospheric phase delay correction in the Los Angeles region by experimental comparison. The StaD decreased by 15.71%, 14.68%, 4.16%, and 13.96% more than the current best GACOS product in each season, respectively.

After comparing the correction performance of various InSAR tropospheric phase delay correction methods, this paper concluded that the GNSS ZTD correction method would be an important means to advance the progress of InSAR tropospheric phase delay correction. Its continuous data at the stations can accurately determine the atmospheric interference to SAR data at the acquisition moment. However, the sparse and uneven distribution of free and publicly available GNSS sites in individual regions will challenge GNSS ZTD spatial interpolation. Therefore, this paper considers that the current solution is to combine GNSS ZTD and satellite remote sensing data to reconstruct high spatial-temporal resolution InSAR tropospheric phase delay maps. Additionally, with the development of GNSS station deployment and public and multi-factor interpolation methods proposed will gradually solve this challenge.

In recent years, many scientists have explored the direction of InSAR tropospheric phase delay correction. However, there is still no universal correction scheme that can eliminate more than 90% of the tropospheric phase delays in the unwrapped phases without space and time constraints. With the launch of more SAR satellites and the free availability of better data sources, more tropospheric/ionospheric information is available to scientists, which will give a strong impetus to the progress of InSAR interferograms atmospheric phase delay removal. At the same time, more scholars can focus on exploring high-precision observations of earth and space based on InSAR, which is undoubtedly essential and urgently needed.

## Figures and Tables

**Table 1:** BP + GNSS model input data correlation and RMSE of output site ZTD and original ZTD.

R Squared between Height and ZTD				ZTD RMSE between GNSS and BP + GNSS			
Date (dd/mm/yyyy)	R^2^	Date (dd/mm/yyyy)	R^2^	Date (dd/mm/yyyy)	RMSE (mm)	Date (dd/mm/yyyy)	RMSE (mm)
18/01/2021	0.997	10/08/2021	0.970	18/01/2021	7.656	10/08/2021	9.207
23/02/2021	0.997	22/08/2021	0.990	23/02/2021	7.729	22/08/2021	7.716
19/03/2021	0.988	15/09/2021	0.991	19/03/2021	8.491	15/09/2021	7.771
12/04/2021	0.991	09/10/2021	0.991	12/04/2021	7.492	09/10/2021	7.569
24/04/2021	0.993	21/10/2021	0.994	24/04/2021	7.644	21/10/2021	6.779
18/05/2021	0.988	14/11/2021	0.997	18/05/2021	8.559	14/11/2021	7.566
11/06/2021	0.993	08/12/2021	0.994	11/06/2021	8.376	08/12/2021	7.386
23/06/2021	0.966	20/12/2021	0.995	23/06/2021	8.397	20/12/2021	7.549
17/07/2021	0.990	-	-	17/07/2021	9.241	-	-

**Table 2:** Statistics for quality assessment of all interferogram corrections.

	Original Unwrapped Phase	Ph-Elev (Linear)	ERA5	MODIS	MERRA2	GACOS	BP + GNSS
Mean of StaD (rad)	4.95	4.12	4.80	4.88	4.48	2.78	2.38
Mean of SDP ^1^ (%)	-	16.81	3.15	1.52	9.58	44.04	52.03
NISD ^2^	42 (all)	42	17	25	21	36	39
CPIN ^3^ (%)	-	100.00	40.48	59.52	50.00	85.71	92.86
Mean of SSIM	-	0.66	0.77	0.79	0.78	0.79	0.78

^1^ StaD decrease percentage (SDP): [(StaD_corrected_- StaD_original_)/StaD_original_] × 100; ^2^ NISD: Number of interferograms for StaD decrease; ^3^ CPIN: Correction percentage of interferogram number.

## Data Availability

All the data used in this research are available upon request by e-mail to the corresponding author.

## Appendix A

**Table A1:** Examples of correction datasets and their characteristics.

Dataset	Description	Data Product	Spatial Resolution	Coverage	Temporal Sampling	Hydrostatic Delay	Wet Delay
GNSS	Propagation delay	Zenith total delay	Station spacing	Global	5 min	Yes	Yes-Including turbulent
GACOS	Operational weather model	Zenith total delay	90 m	Global	1 min	Yes	Yes-Including turbulent
ERA5	Weather reanalysis model	Pressure; temperature; humidity	0.25°	Global	1 h	Yes	Yes-Including turbulent
MERRA2	Weather reanalysis model	Pressure; temperature; humidity	0.50° × 0.625°	Global	3 h	Yes	Yes-Including turbulent
NARR	Weather reanalysis model	Pressure; temperature; humidity	32 km (0.3°)	US only	3 h	Yes	Yes-Including turbulent
WRF	Weather model	Pressure; temperature; humidity	adjustable	Global	adjustable	Yes	Yes-Including turbulent
MODIS	Spectroradiometer	Water vapor	1 km	Global	1–2 day	No	Yes-Including turbulent
MERIS	Spectrometer	Water vapor	1.2 km	Global	1 day (17 May 2002–4 August 2012)	No	Yes-Including turbulent
OLCI	Radiometer	Water vapor	300 m	Global	1–2 day	No	Yes-Including turbulent
Power–law correction	Spatially varying troposphere	Ph-Elev (Phase/DEM)	Same as the SAR data	Global	Same as the SAR data	Yes	Yes- no turbulence
Linear correction	Uniform troposphere	Ph-Elev (Phase/DEM)	Same as the SAR data	Global	Same as the SAR data	Yes	Yes- no turbulence
